# Hypoglycaemia and Cardiac Arrhythmias in Type 1 Diabetes Mellitus: A Mechanistic Review

**DOI:** 10.3390/jpm16010045

**Published:** 2026-01-09

**Authors:** Kyriaki Mavromoustakou, Christos Fragoulis, Kyriaki Cholidou, Zoi Sotiropoulou, Nektarios Anagnostopoulos, Ioannis Gastouniotis, Stavroula-Panagiota Lontou, Kyriakos Dimitriadis, Anastasia Thanopoulou, Christina Chrysohoou, Konstantinos Tsioufis

**Affiliations:** 1 First Cardiology Clinic, School of Medicine, National and Kapodistrian University of Athens, Hippokration General Hospital, 114 Vas. Sofias Ave., 11527 Athens, Greece; dimitriadiskyr@yahoo.gr (K.D.); chrysohoou@usa.net (C.C.); ktsioufis@hippocratio.gr (K.T.); 2 1st Respiratory Department, National and Kapodistrian University of Athens, Sotiria Chest Diseases Hospital, 11527 Athens, Greece; kg.cholidou@yahoo.gr (K.C.); zoisotiropoulou96@gmail.com (Z.S.); aris.anag@yahoo.gr (N.A.); 3 Diabetes Center, 2nd Department of Internal Medicine, Medical School, National and Kapodistrian University of Athens, Hippokration General Hospital of Athens, 11527 Athens, Greece; giannisgast@gmail.com (I.G.); a_thanopoulou@hotmail.com (A.T.); 4 First Department of Internal Medicine, National and Kapodistrian University of Athens, Laiko General Hospital, 11527 Athens, Greece; slondou@gmail.com

**Keywords:** type 1 diabetes mellitus, hypoglycaemia, cardiac arrhythmia, QTc prolongation, atrial fibrillation, autonomic dysfunction, impaired awareness of hypoglycaemia, dead-in-bed syndrome, continuous glucose monitoring

## Abstract

Hypoglycaemia in patients with type 1 diabetes mellitus (T1DM) remains a major clinical burden and, beyond its metabolic complications, can cause serious cardiac arrhythmias. Multiple mechanisms lead to different types of arrhythmias during hypoglycaemia. However, existing studies often involve mixed diabetes populations, small cohorts, or limited monitoring during nocturnal periods, leaving a critical gap in understanding the links between glucose fluctuations and arrhythmic events. This review provides an updated combination of experimental and clinical evidence describing how autonomic dysfunction and ionic imbalances lead to electrophysiological instability and structural remodelling of the myocardium during hypoglycaemia. Continuous glucose monitoring (CGM) combined with electrocardiographic or wearable rhythm tracking may enable early detection of glycemic and cardiac disturbances and help identify high-risk individuals. Future prospective studies using combined CGM–ECG monitoring, particularly during sleep, are essential to clarify the relationship between hypoglycaemia and arrhythmic events.

## 1. Introduction

In 2025, approximately 9.5 million people worldwide had a confirmed diagnosis of type 1 diabetes mellitus (T1DM) [1]. T1DM is associated with an increased risk of complications, with chronic hyperglycaemia leading to long-term microvascular and macrovascular damage, while hypoglycaemia causing plethora of acute morbidities [2]. These include cognitive impairment, ranging from confusion to coma, physical injury, and cardiovascular effects, such as arrhythmias, highlighting their serious impact on patient safety and quality of life [3].

The relationship between hypoglycaemia and arrhythmia risk in patients with T1DM has been studied, and multiple studies have demonstrated this association. Qtc prolongation is the most frequent complication followed by premature atrial and ventricular contractions, atrioventricular block, and atrial fibrillation [4].

Dead-in-bed syndrome (DIB) is a rare phenomenon in which severe nocturnal hypoglycaemia in patients with T1DM causes death during sleep without prior symptoms. This phenomenon is possibly connected to malignant cardiac arrhythmias induced by hypoglycaemia [5,6].

Several studies have reported associations between obstructive sleep apnea (OSA), T1DM, and cardiac arrhythmias [7,8]. T1DM increases the risk of OSA through mechanisms such as glycemic variability and autonomic neuropathy, whereas OSA exacerbates glycemic instability via intermittent hypoxia. Intermittent hypoxia may also trigger atrial and ventricular arrhythmias, especially during nocturnal periods [9,10]. However, the precise risk of cardiac arrhythmias in patients who have both OSA and T1DM is unknown because no study has directly investigated this three-way relationship.

This review aims to comprehensively summarize experimental, clinical, and real-world evidence on the mechanisms and pathophysiological pathways linking hypoglycaemia to cardiac arrhythmias specifically in patients with type 1 diabetes mellitus. To our knowledge, this is the first comprehensive mechanistic review focused exclusively on type 1 diabetes to include experimental, clinical, and real-world simultaneous glucose and cardiac rhythm monitoring evidence.

## 2. Materials and Methods

This article is a narrative review that is based on structured literature searches to support the identification and synthesis of existing evidence on the mechanisms of arrhythmias during hypoglycaemia in patients with T1DM. The review does not constitute a formal systematic review or meta-analysis, and no protocol was registered.

### 2.1. Search Strategy

A review of the literature was conducted to examine the mechanisms of arrhythmias during hypoglycaemia in patients with T1DM. The search was carried out on Medline, PubMed, EMBASE, Web of Science, Cochrane Central Register of Controlled Trials, and reference lists of retrieved reports using the keywords “T1DM”, “hypoglycaemia”, “cardiac arrhythmia”, “atrial fibrillation”, “QTc prolongation”, and “OSA” in combination with the Boolean operators AND or OR. Only articles published in English from 2005 onwards were included, with the most recent search conducted in August 2025.

An AI-based language tool was used solely for grammatical improvements and the generation of illustrative elements in selected diagrams. No scientific content, interpretation, or conclusions were generated by the AI.

### 2.2. Study Selection

Studies were eligible for inclusion if they involved participants with type 1 diabetes mellitus (T1DM) exclusively. Eligible articles examined the occurrence of cardiac arrhythmias during hypoglycemia in individuals with T1DM, reported the prevalence or incidence of arrhythmias in T1DM cohorts, or investigated the association between obstructive sleep apnea (OSA), T1DM, and cardiac arrhythmias. Reviews, editorials, and commentaries were excluded, as were studies that did not provide sufficient data for analysis. Only studies published in English were considered.

### 2.3. Data Extraction

Two reviewers (KM and CF) independently extracted data from all eligible original studies or meta-analyses and systemic reviews and cross-checked entries, using classified forms. We assessed study quality using established tools (NOS/ROBINS-I/JBI). Where applicable, the GRADE approach was used to evaluate the certainty of evidence for major outcomes. The initial search retrieved 144 articles. After removal of duplicates and screening of titles and abstracts, 102 full-text articles were assessed for relevance. Of these, 67 studies were included in the final review.

## 3. Pathophysiological Mechanisms of Hypoglycemia-Induced Arrhythmias in Type 1 Diabetes Mellitus

Hypoglycaemia is the main obstacle to achieving glycemic control in the vast majority of patients with type 1 diabetes mellitus (T1DM) [11]. Repeated insulin-induced hypoglycaemia decreases the sympathoadrenal response, primarily reducing epinephrine secretion, and lowers the glycemic threshold that triggers these defensive mechanisms. This creates a vicious cycle in which hypoglycaemia leads to impaired autonomic responses, thereby increasing the risk of subsequent hypoglycaemic episodes [12,13].

To adapt to repeated hypoglycaemia, the brain increases the expression of glucose transporters and shifts its energy metabolism to utilize alternative substrates such as lactate [14,15]. This central adaptation also involves enhanced inhibitory gamma-aminobutyric acid (GABA) signalling in the ventromedial hypothalamus (VMH), which suppresses the autonomic nervous system activation essential for counter-regulatory responses during hypoglycaemia. Elevated GABAergic tone inhibits the release of counter-regulatory hormones, including epinephrine, which further decreases the body’s ability to restore normal glucose levels. Moreover, exercise and nocturnal hypoglycaemia further impair these counter-regulatory responses and decrease symptom recognition, compounding the risk of severe hypoglycaemia [16,17].

Together, these mechanisms contribute to the development and progression of Hypoglycemia-Associated Autonomic Failure (HAAF), a condition marked by defective glucose counter-regulation and impaired symptom awareness, significantly increasing the risk of severe hypoglycaemic events in patients with T1DM [16].

### 3.1. Atrial Fibrillation

More clinical evidence links hypoglycaemia in patients with T1DM. Severe hypoglycemic events play a significant role in the initiation, maintenance, and progression of atrial fibrillation (AF). The autonomic nervous system (ANS), comprising the sympathetic and parasympathetic branches, influences cardiac electrophysiology and atrial structure, thereby affecting AF dynamics. Therefore, hypoglycaemia-induced autonomic imbalance represents one of the major mechanisms which link glycaemic instability and AF development in T1DM [18].

#### 3.1.1. Sympathetic System

Strong experimental evidence supporting the arrhythmogenic role of hypoglycaemia-induced sympathetic activation comes from animal models. Reno et al. [19] demonstrated that severe hypoglycaemia can cause lethal cardiac arrhythmias via sympathoadrenal activation. In their study, insulin-induced hypoglycaemia in rodents led to marked increases in circulating catecholamines, QT prolongation, and malignant arrhythmias, effects that were significantly attenuated by adrenergic blockade or adrenalectomy [19]. From a clinical and epidemiological perspective, long-term observational data further support the role of autonomic dysfunction in AF development. A 20-year follow-up study demonstrated that markers of cardiac autonomic dysfunction were independently associated with incident AF [20]. Importantly, this association persisted after adjustment for cardiovascular risk factors, suggesting a direct pathophysiological role of autonomic mechanisms. Finally, in T1DM populations, meta-analytic data indicate that individuals with T1DM have a significantly elevated risk of cardiovascular disease compared with the general population [21]. While AF is not always analyzed as a discrete endpoint, the increased burden of arrhythmogenic risk factors supports the plausibility that hypoglycaemia caused by sympathetic stimulation contribute to AF development in this population.

**Figure 1:**
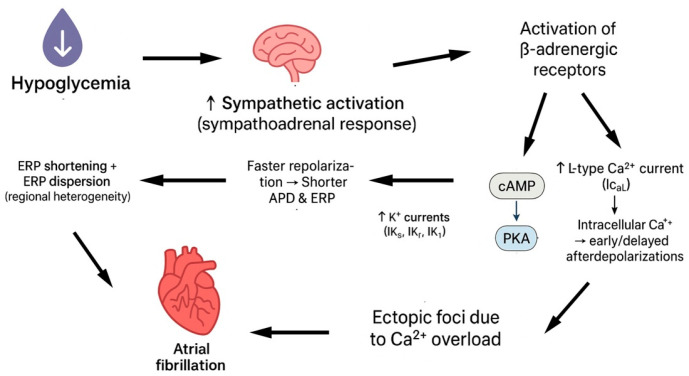
Hypoglycaemia leads to atrial fibrillation via sympathetic stimulation.

Sympathetic stimulation shortens the effective refractory period (ERP) of atrial tissue, which is the period during which atrial cells cannot be re-excited after an action potential. Acute hypoglycaemia triggers a sympathoadrenal surge, resulting in elevated circulating catecholamines that directly influence atrial electrophysiology. The mechanism behind that is the activation of β-adrenergic receptors, leading to cAMP/PKA-mediated phosphorylation of ion channels. This increases the potassium currents responsible for repolarization, accelerating repolarization, shortening the action potential duration, and thus reducing the refractory period. Specifically, PKA phosphorylation augments delayed-rectifier K^+^ currents, increasing outward current during the plateau and terminal repolarization phases. The net effect is a faster repolarization, a shorter APD, and, therefore, a reduced ERP. Because ERP is the time window during which excitable tissue cannot be re-activated, shortening of ERP allows premature electrical impulses to propagate through atrial tissue before it has fully recovered, increasing the chance of re-entrant circuits [19,20]. Furthermore, sympathetic activity can cause dispersion, which refers to differences in refractory periods across various regions of the atria, due to uneven receptor distribution. When some areas recover faster than others, it creates electrical heterogeneity. This heterogeneity can cause wave breaks in the propagation of electrical impulses, facilitating re-entry and AF. Finally, the activation of the β-adrenergic receptor enhances calcium influx via L-type calcium channels, increasing intracellular calcium. Elevated intracellular calcium can lead to early or delayed afterdepolarizations, creating ectopic foci that can initiate AF [20,21] (Figure 1).

#### 3.1.2. Parasympathetic System

Parasympathetic impairment induced by hypoglycaemia in T1DM has been provided by Koivikko et al. [22], who investigated the effects of sustained insulin-induced hypoglycaemia on cardiovascular autonomic regulation. In this controlled clinical study, hypoglycaemia was associated with a marked reduction in indices of parasympathetic activity, including reduced heart rate variability and impaired vagal reflexes. These findings demonstrate that hypoglycaemia suppresses parasympathetic modulation, even in the absence of structural heart disease [22].

**Figure 2:**
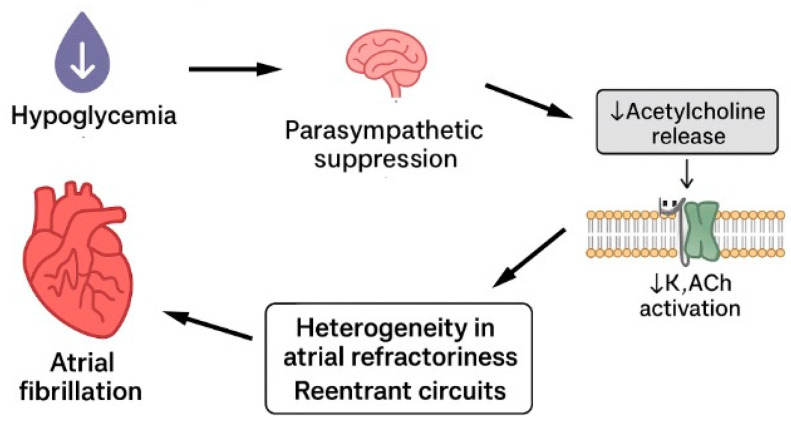
Hypoglycaemia leads to atrial fibrillation via suppression of parasympathetic system.

Repeated hypoglycaemia leads to suppression of parasympathetic activity. Chronic hypoglycaemia and HAAF are associated with impaired vagal modulation of cardiac electrophysiology. The parasympathetic system, through acetylcholine release, activates acetylcholine-sensitive potassium channels in atrial myocytes, exerting a stabilizing influence on atrial electrophysiology by slowing the heart rate, prolonging atrial refractoriness, and counterbalancing sympathetic effects (IK,ACh) [22]. Dysfunction of this system can lead to altered regulation of heart rate and electrical conduction in the atria. This creates heterogeneity in atrial refractoriness and promotes the formation of re-entrant circuits. Such electrical heterogeneity and instability increase the risk of atrial fibrillation (AF) initiation and maintenance. Furthermore, chronic autonomic neuropathy resulting from diabetes may exacerbate parasympathetic dysfunction. Hypoglycemia-induced autonomic failure can further impair vagal tone, compounding electrical instability in the atria and creating a vicious cycle [4] (Figure 2).

#### 3.1.3. Atrial Remodelling

Autonomic imbalance due to repeated hypoglycaemia can cause structural remodelling in atrial tissue, especially atrial fibrosis. Chronic metabolic stress, oxidative injury, and autonomic dysregulation in T1DM contribute to myocardial fibrotic remodelling. Type 1 diabetes mellitus (T1DM) has been correlated with cardiac fibrosis [23]. Histopathological evidence has long supported a link between T1DM and myocardial fibrosis. Endomyocardial biopsy studies have demonstrated increased interstitial fibrosis in patients with diabetes and impaired cardiac function, even in the absence of coronary artery disease [23]. These findings suggest that diabetes itself can directly induce structural myocardial changes, providing a substrate for atrial electrical instability [23].

Structural remodelling includes the development of fibrosis and changes in the length of heart muscle cells caused by the heart’s enlargement. This dilation increases resistance along the length of the muscle cells, which worsens the electrical conduction problems seen in patients with diabetic cardiomyopathy [24]. Structural remodelling, particularly atrial fibrosis, is a major factor contributing to atrial fibrillation (AF). This occurs through two mechanisms: re-entrant activity and enhanced automaticity. Fibrotic tissue is unexcitable, which can cause electrical waves to circulate around it, leading to re-entry circuits. This disruption can result in chaotic conduction patterns that increase the likelihood of AF. Additionally, fibrosis can trigger ectopic activity by impairing electrical coupling between heart cells, allowing abnormal after-depolarizations. Animal models have confirmed that fibrosis increases conduction heterogeneity and automaticity, amplifying the arrhythmic substrate in the atria [25].

The way fibrosis affects electrical conduction in the atria is not just about the amount of fibrosis, but more about how it disrupts the connections between heart muscle cells [26,27]. Fibrosis thickens the collagen layers between muscle fibres, particularly interfering with the side-to-side connections that help coordinate the heart’s electrical signals. This results in uneven and slowed electrical conduction, making the atria more prone to abnormal circuits that can trigger AF. Additionally, fibrosis alters the arrangement and connectivity of muscle fibres in different regions of the atria—such as between the outer (epicardial) and inner (endocardial) layers—leading to blocks in electrical signals and a lack of synchronization between these layers. Both clinical observations and animal studies have found that fibrosis is linked to slower, more chaotic electrical activity in the atria, increasing the complexity and persistence of AF, which underscores its key role in maintaining the arrhythmia [28,29].

The precise mechanisms by which different types of fibrosis lead to arrhythmogenesis require further investigation and are not yet completely understood, especially in patients with T1DM. There are limited studies on atrial fibrosis in patients with T1DM, particularly in humans.

#### 3.1.4. Future Research Directions

Future investigations should further investigate autonomic framework to clarify how hypoglycaemia-induced sympathetic overactivation and parasympathetic withdrawal contribute to atrial electrophysiological instability in patients with T1DM. While experimental and epidemiological studies have linked sympathetic activation and impaired vagal modulation to arrhythmogenesis, their interaction during and after hypoglycemic episodes remain poorly defined. Longitudinal clinical studies combining continuous glucose monitoring with detailed autonomic assessment could help establish the relationship between glycaemic instability, autonomic imbalance, and AF onset [4,18,19,20,21,22].

Structural consequences of chronic metabolic and autonomic stress in patients with T1DM also require larger research studies. Although myocardial fibrosis has been documented in diabetes, data specifically characterizing atrial fibrosis in this population remain limited, especially in humans [23]. Advanced imaging and high-resolution electrophysiological mapping studies are needed to define microstructural organization of atrial fibrosis and its relationship to conduction disturbances [24,25,26,27,28,29].

### 3.2. QTc Prolongation

The QT interval represents the beginning of the QRS complex, which is the start of ventricular depolarization, to the end of ventricular repolarization. It reflects the whole activity of multiple cardiac ion channels, including inward sodium and calcium currents and outward potassium currents. Prolongation of the QT interval indicates delayed ventricular repolarization and can occur due to a variety of causes, such as genetic factors, electrolyte abnormalities, medications, autonomic nervous system imbalance, metabolic disorders, and ischemic heart disease [30].

Excessive QTc prolongation predisposes to early afterdepolarizations (EADs), which can trigger malignant arrhythmias such as polymorphic ventricular tachycardia and torsades de pointes. Importantly, arrhythmic risk does not depend only on absolute QTc duration but also on repolarization heterogeneity, often reflected by QT dispersion [30].

Epidemiological data from large T1DM cohorts show that frequent/severe hypoglycaemia is independently associated with a greater prevalence and incidence of QTc prolongation [30]. During hypoglycaemia, the acute decrease in blood glucose activates the sympathoadrenal response, resulting in elevated levels of epinephrine and norepinephrine in the blood. Circulating catecholamines bind to β-adrenergic receptors on ventricular myocytes, activating the cAMP/PKA pathway, which increases L-type calcium current, prolongs the plateau phase of the action potential, and enhances intracellular calcium loading, thereby promoting delayed afterdepolarizations [11,12]. Moreover, the same pathway stimulates the Na^+^/K^+^-ATPase in skeletal muscle, driving K^+^ into the cells and resulting in acute hypokalemia. The reduction in extracellular [K^+^] decreases the driving force for K^+^ efflux, slows phase 3 repolarization, prolongs ventricular action potential duration, and increases the QT interval on the ECG [31,32].

**Figure 3:**
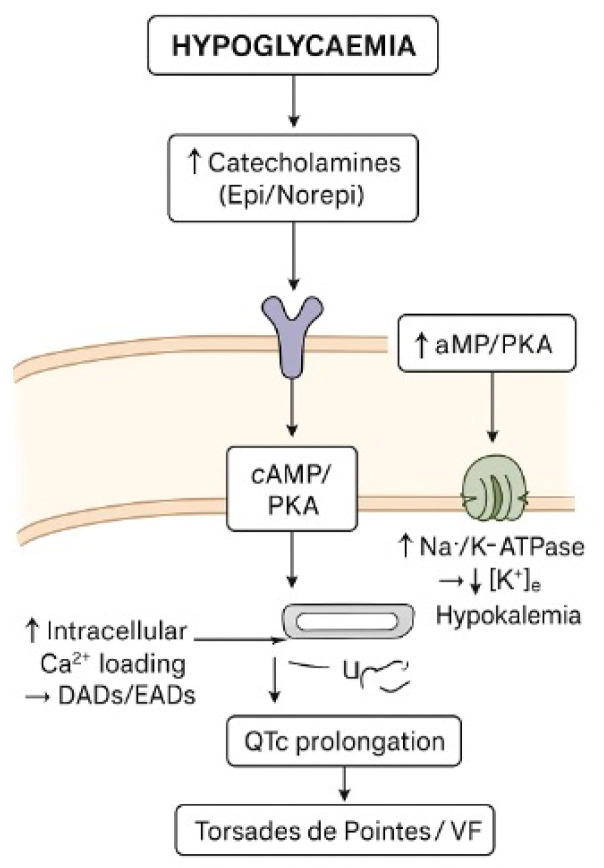
Diagram of how hypoglycaemia cause QTc prolongation.

The combination of increased inward calcium currents and decreased outward potassium currents enhances heterogeneity of repolarization across different regions of the ventricle, leading to increased QT dispersion and a higher propensity for early afterdepolarizations (EADs), which can trigger malignant arrhythmias such as polymorphic ventricular tachycardia or torsades de pointes. Thus, QTc prolongation during hypoglycaemia results from a complex interplay of neurohormonal, ionic, and cellular mechanisms that together increase the risk of malignant ventricular arrhythmias in individuals with type 1 diabetes [11] (Figure 3).

### 3.3. Bradycardia and Atrioventricular Block

In patients with T1DM, severe and prolonged hypoglycaemia, especially during sleep and in individuals with autonomic dysfunction, can lead to parasympathetic dominance. The vagus nerve becomes dominant, resulting in sinus bradycardia, atrioventricular block, and sometimes asystole [33].

Specifically, during hypoglycaemia ATP falls due to glucose is one of the main substances for ATP generation. As a result of ATP reduction, there is not enough energy for membrane ion pumps. Even the slightest reduction impairs the pumps’ function. One of the main pumps which plays an important role in the conduction system of heart is the Na^+^/K^+^ pump (3 Na^+^ out/2 K^+^ in per ATP), which is energy-dependent. As a result of ATP depletion, the intracellular sodium concentration increases, causing a reduction in transmembrane Na^+^ gradient. This decreases action potential upstroke velocity (dV/dt), slowing the conduction velocity in atrial/ventricular muscle [34]. Moreover, when the intracellular sodium rises, the Na^+^/Ca^2+^ (3 Na^+^ in/1 Ca^2+^ out) exchanger (NCX) can operate less effectively in forward mode or even reverse, causing an increase in the intracellular Ca^2+^. Persistent Ca^2+^ elevation activates non-selective cation currents (Iti) and shifts resting membrane potential upward (less negative). SA and AV nodal cells become partially depolarized (≈–50 mV instead of –60 mV). At this voltage, most fast Na^+^ channels (and some Ca^2+^ channels) are inactivated, causing slower upstroke and slower conduction, which lead to bradycardia and AV block. Furthermore, sustained Ca^2+^ elevation causes Ca^2+^-dependent inactivation of L-type Ca^2+^ channels. Since AV nodal conduction and SA node automaticity depend heavily on ICa,L, this inactivation directly slows pacemaking and conduction [35].

**Figure 4:**
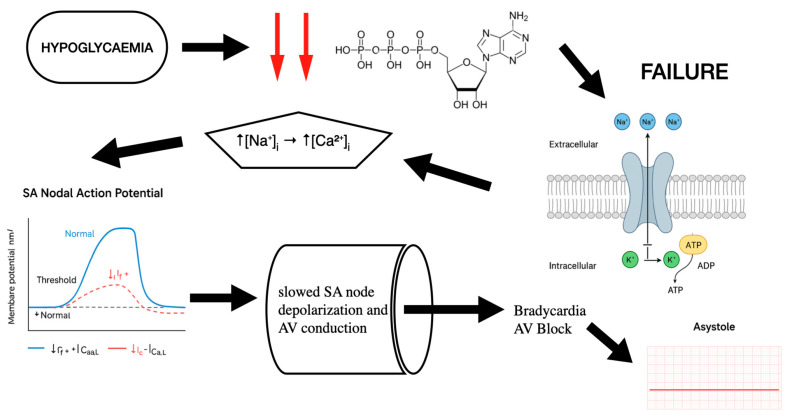
Mechanistic analysis: how hypoglycaemia leads to bradycardia and AV block.

Finally, SERCA (sarco/endoplasmic reticulum Ca^2+^-ATPase) needs ATP to sequester cytosolic Ca^2+^ into the SR. ATP inefficiency slows SERCA, increasing cytosolic Ca^2+^ and promoting diastolic Ca^2+^ leakage. Cytosolic Ca^2+^ overload and ATP depletion inhibit cyclic-nucleotide-gated channels that carry If (funny current). Reduced If causes slower diastolic depolarization which leads to sinus bradycardia [36] (Figure 4).

It is important to note that while hypoglycaemia-associated arrhythmias have been directly documented in patients with T1DM through ECG monitoring, many of the intracellular ionic and metabolic mechanisms described here are derived from experimental electrophysiology and animal models studies. Direct measurement of myocardial ATP levels, ion gradients, or calcium handling during spontaneous hypoglycaemia in humans is not currently possible. These mechanisms, therefore, represent a biologically plausible framework that combines experimental data with observed clinical data in T1DM.

### 3.4. “Dead in Bed” Syndrome

Qtc prolongation is strongly connected with “Dead-in-Bed” syndrome, a sudden cardiac death in young individuals with T1DM. It describes unexpected death during sleep, often without any obvious cause found after necropsy. Sudden unexpected death during sleep is approximately ten times more frequent in individuals with type 1 diabetes. First recognized in the 1990s, it has since been consistently reported in epidemiological studies as a rare complication of T1DM [37].

Severe and recurrent episodes of hypoglycaemia during sleep in people with T1DM can lead to QTc prolongation with mechanisms that we explained before, as well as bradycardia and atrioventricular block. Evidence links nocturnal severe hypoglycaemia, especially in people with impaired awareness of hypoglycaemia (IAH), to fatal cardiac arrhythmias as a plausible final pathway. IAH is a reduction or loss of autonomic warning symptoms to hypoglycaemia and significantly increases the risk of severe hypoglycaemia. When hypoglycaemia occurs during sleep in someone with IAH, the usual protective awakening and counter-regulatory responses may be blunted, causing prolonged hypoglycaemia that can trigger the electrophysiologic changes described above [38].

Emerging continuous monitoring technologies, such as integrated continuous glucose monitoring (CGM) combined with smartwatch’s photoplethysmography, implantable loop recorders (ILRs), or patch-based ECG sensors, allow detection of glycemic fluctuations and cardiac arrhythmias simultaneously. Utilizing these tools, with automated insulin delivery systems and personalized hypoglycaemia alarms, can detect nocturnal hypoglycaemia early and potentially prevent malignant arrhythmias during sleep [39,40].

The current understanding of hypoglycaemia-induced arrhythmias in type 1 diabetes is limited by several challenges. Because severe arrhythmic events, such as those leading to sudden death during sleep, are rare, gathering large and definitive clinical datasets is difficult. Furthermore, teasing apart direct cause-and-effect relationships is complicated by the presence of multiple overlapping factors, including autonomic nervous system dysfunction and conditions like obstructive sleep apnea. While experimental and observational research offers valuable mechanistic insights, there remains a scarcity of human studies on key aspects like atrial fibrosis and nocturnal arrhythmias. Emerging technologies that combine continuous glucose monitoring with cardiac rhythm tracking offer promising avenues for future research but require further validation in this patient group. This highlights the need for caution in interpreting current findings and emphasizes opportunities for continued investigation [41].

## 4. Personalized Medicine

This review highlights the importance of personalized medicine in the management of patients with T1DM to prevent lethal episodes of cardiac arrhythmias. Individual physiological vulnerabilities play a crucial role in the severity of these episodes. Impaired awareness of hypoglycaemia, autonomic neuropathy, nocturnal glycemic patterns, electrolyte responses, and coexisting conditions such as obstructive sleep apnea are factors which contribute to creation of arrhythmic phenotypes among individuals with T1DM. Recognizing these high-risk patients underscores the need for closer glycemic monitoring and reconsider glycemic targets, taking into consideration risk stratification that incorporates autonomic function, cardiac electrophysiology, and hypoglycaemia exposure.

Personalized therapeutic strategies can combine the use of continuous glucose monitoring (CGM) with electrocardiographic or wearable rhythm surveillance, allowing real-time identification of high-risk glycemic–electrophysiological interactions. This monitoring approach will help investigate the correlation between hypoglycaemic episodes and cardiac rhythm disturbances, particularly during nocturnal periods when symptoms often go unrecognized. Moreover, this intensive monitoring can include personalized alarm thresholds based on patients’ data for optimal glycemic control and to prevent arrhythmias.

Finally, future datasets from CGM and ECG wearables can be utilized for the creation of risk prediction models and for phenotyping autonomic dysfunction to identify patients at the highest risk for fatal arrhythmias. This could drastically change the follow-up strategies, device selection, and therapeutic targets for high-risk patients. Hence, personalized medicine appears to be a vital evolution in the cardiovascular care of patients with T1DM.

## 5. Conclusions

Hypoglycaemia, especially recurrent episodes, in patients with T1DM is critical and can trigger fatal arrhythmias. Repeated hypoglycaemia can cause autonomic disturbances that contribute to electrical heterogeneity, QTc prolongation, bradyarrhythmia, and atrial fibrillation (Table 1).

Despite increasing evidence linking hypoglycaemia to malignant arrhythmias, the precise interaction between glycemic variability, autonomic impairment, and cardiac electrical instability remain incompletely understood.

Future research should focus on prospective studies employing continuous glucose and cardiac rhythm monitoring, particularly during sleep, to clarify the temporal relationship between hypoglycaemia and arrhythmia onset. It is critical to identify high-risk patients for hypoglycaemia-induced arrhythmias to reduce morbidity and mortality among individuals with T1DM.

**Figure 5:**
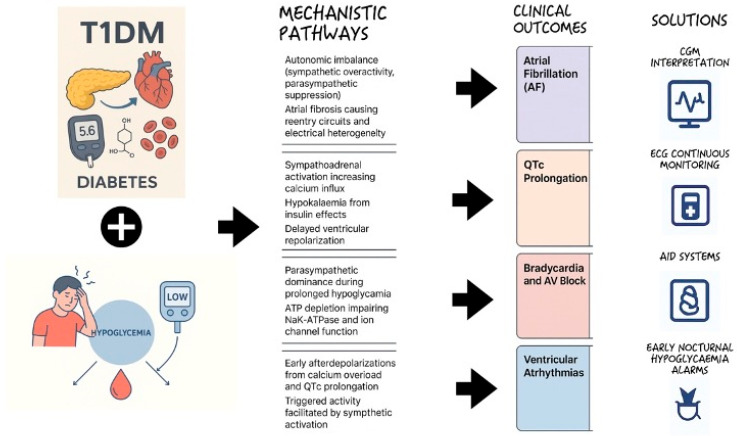
Hypoglycaemia-induced cardiac arrhythmias in type 1 diabetes mellitus: mechanistic overview.

Polysomnography combined with CGM and ECG monitoring could contribute to understanding patterns of nocturnal hypoglycaemia and correlate them with arrhythmic events. This would help identify high-risk individuals and guiding targeted interventions to mitigate hypoglycaemia-related cardiovascular risk in T1DM (Figure 5).

## Figures and Tables

**Table 1:** Pathophysiological mechanisms of hypoglycemia-induced arrhythmias in type 1 diabetes mellitus. AF; atrial fibrillation, OSA; obstructive sleep apnea, IAH; impaired awareness of hypoglycaemia.

Arrhythmia Type	Mechanisms	Associated Risk Factors
Atrial Fibrillation (AF)	Autonomic imbalance (sympathetic overactivity, parasympathetic suppression), atrial fibrosis causing re-entry circuits and electrical heterogeneity	Recurrent hypoglycaemia, autonomic neuropathy, atrial fibrosis, OSA
QTc Prolongation	Sympathoadrenal activation increasing calcium influx, hypokalemia from insulin effects, delayed ventricular repolarization	Frequent/severe hypoglycaemia, glycemic variability, longer diabetes duration, poor glycemic control
Bradycardia and AV Block	Parasympathetic dominance during prolonged hypoglycaemia, ATP depletion impairing NaK-ATPase and ion channel function causing slowed conduction and pacemaker suppression	Nocturnal hypoglycaemia, autonomic dysfunction, Impaired awareness of hypoglycaemia (IAH)
Ventricular Arrhythmias	Early afterdepolarizations from calcium overload and QTc prolongation, triggered activity facilitated by sympathetic activation	Severe hypoglycaemic events, cardiac autonomic neuropathy, underlying structural heart changes
Dead-in-Bed Syndrome	Combination of nocturnal hypoglycaemia, impaired autonomic responses, malignant arrhythmias including bradyarrhythmia and QT prolongation	Nocturnal severe hypoglycaemia, IAH, impaired cardiac autonomic regulation

## Data Availability

The original contributions presented in this study are included in the article. Further inquiries can be directed to the corresponding authors.

## Abbreviations

The following abbreviations are used in this manuscript:

T1DM	Type 1 diabetes mellitus
CGM	Continuous glucose monitoring
DIB	Dead-in-bed
OSA	Obstructive sleep apnea
GABA	Gamma-aminobutyric acid
VMH	Ventromedial hypothalamus
HAAF	Hypoglycemia-Associated Autonomic Failure
AF	Atrial fibrillation
ANS	Autonomic nervous system
ERP	Effective refractory period
EADs	Early afterdepolarizations
SERCA	Sarco/endoplasmic reticulum Ca^2+^-ATPase
IAH	Impaired awareness of hypoglycaemia
ILRs	Implantable loop recorders

## References

[1] Ogle G.D., Wang F., Haynes A., Gregory G.A., King T.W., Deng K., Dabelea D., James S., Jenkins A.J., Li X. (2025). Global type 1 diabetes prevalence, incidence, and mortality estimates 2025: Results from the International diabetes Federation Atlas, 11th Edition, and the T1D Index Version 3.0. Diabetes Res. Clin. Pract..

[2] Longendyke R., Grundman J.B., Majidi S. (2024). Acute and Chronic Adverse Outcomes of Type 1 Diabetes. Endocrinol. Metab. Clin. N. Am..

[3] Amiel S.A. (2021). The consequences of hypoglycaemia. Diabetologia.

[4] Andersen A., Jørgensen P.G., Knop F.K., Vilsbøll T. (2020). Hypoglycaemia and cardiac arrhythmias in diabetes. Ther. Adv. Endocrinol. Metab..

[5] Clark A.L., Best C.J., Fisher S.J. (2014). Even silent hypoglycemia induces cardiac arrhythmias. Diabetes.

[6] Poudel R.R., Kafle N.K., Belbase B., Kafle P.K. (2015). Dead in bed syndrome: Mystery and fear. J. Soc. Health Diabetes.

[7] Alshehri Z., Subramanian A., Adderley N.J., Gokhale K.M., Karamat M.A., Ray C.J., Kumar P., Nirantharakumar K., Tahrani A.A. (2022). Risk of incident obstructive sleep apnoea in patients with type 1 diabetes: A population-based retrospective cohort study. Diabetologia.

[8] Menon T., Ogbu I., Kalra D.K. (2024). Sleep-Disordered Breathing and Cardiac Arrhythmias. J. Clin. Med..

[9] Farabi S.S. (2016). Type 1 Diabetes and Sleep. Diabetes Spectr..

[10] Framnes S.N., Arble D.M. (2018). The Bidirectional Relationship Between Obstructive Sleep Apnea and Metabolic Disease. Front. Endocrinol..

[11] Cryer P.E. (2008). The barrier of hypoglycemia in diabetes. Diabetes.

[12] Cryer P.E. (2013). Mechanisms of hypoglycemia-associated autonomic failure in diabetes. N. Engl. J. Med..

[13] Cryer P.E. (2005). Mechanisms of hypoglycemia-associated autonomic failure and its component syndromes in diabetes. Diabetes.

[14] Wiegers E.C., Rooijackers H.M., Tack C.J., Heerschap A., de Galan B.E., van der Graaf M. (2016). Brain lactate concentration falls in response to hypoglycemia in patients with type 1 diabetes and impaired awareness of hypoglycemia. Diabetes.

[15] Stanley S., Moheet A., Seaquist E.R. (2019). Central mechanisms of glucose sensing and counterregulation in defense of hypoglycemia. Endocr. Rev..

[16] Hedrington M.S., Farmerie S., Ertl A.C., Wang Z., Tate D.B., Davis S.N. (2010). Effects of antecedent GABAA activation with alprazolam on counterregulatory responses to hypoglycemia in healthy humans. Diabetes.

[17] Galassetti P., Tate D., Neill R.A., Richardson A., Leu S.-Y., Davis S.N. (2006). Effect of differing antecedent hypoglycemia on counterregulatory responses to exercise in type 1 diabetes. Am. J. Physiol. Endocrinol. Metab..

[18] Chen P.S., Chen L.S., Fishbein M.C., Lin S.F., Nattel S. (2014). Role of the autonomic nervous system in atrial brillation: Pathophysiology and therapy. Circ. Res..

[19] Reno C.M., Daphna-Iken D., Chen Y.S., VanderWeele J., Jethi K., Fisher S.J. (2013). Severe hypoglycemia-induced lethal cardiac arrhythmias are mediated by sympathoadrenal activation. Diabetes.

[20] Agarwal S.K., Norby F.L., Whitsel E.A., Soliman E.Z., Chen L.Y., Loehr L.R., Fuster V., Heiss G., Coresh J., Alonso A. (2017). Cardiac Autonomic Dysfunction and Incidence of Atrial Fibrillation: Results from 20 Years Follow-Up. J. Am. Coll. Cardiol..

[21] Cai X., Li J., Cai W., Chen C., Ma J., Xie Z., Dong Y., Liu C., Xue R., Zhao J. (2021). Meta-analysis of type 1 diabetes mellitus and risk of cardiovascular disease. J. Diabetes Complicat..

[22] Koivikko M.L., Salmela P.I., Airaksinen K.J., Tapanainen J.S., Ruokonen A., Makikallio T.H., Huikuri H.V. (2005). Effects of sustained insulin-induced hypoglycemia on cardiovascular autonomic regulation in type 1 diabetes. Diabetes.

[23] Sutherland C.G.G., Fisher B.M., Frier B.M., Dargie H.J., More I.A.R., Lindop G.B.M. (1989). Endomyocardial biopsy pathology in insulin-dependent diabetic patients with abnormal ventricular function. Histopathology.

[24] Aromolaran A.S., Boutjdir M. (2017). Cardiac Ion Channel Regulation in Obesity and the Metabolic Syndrome: Relevance to Long QT Syndrome and Atrial Fibrillation. Front. Physiol..

[25] Karakasis P., Theofilis P., Vlachakis P.K., Korantzopoulos P., Patoulias D., Antoniadis A.P., Fragakis N. (2024). Atrial Fibrosis in Atrial Fibrillation: Mechanistic Insights, Diagnostic Challenges, and Emerging Therapeutic Targets. Int. J. Mol. Sci..

[26] Korantzopoulos P., Letsas K.P., Tse G., Fragakis N., Goudis C.A., Liu T. (2018). Inflammation and Atrial Fibrillation: A Comprehensive Review. J. Arrhythmia.

[27] Verheule S., Schotten U. (2021). Electrophysiological Consequences of Cardiac Fibrosis. Cells.

[28] Maesen B., Verheule S., Zeemering S., La Meir M., Nijs J., Lumeij S., Lau D.H., Granier M., Crijns H.J., Maessen J.G. (2022). Endomysial Fibrosis, Rather than Overall Connective Tissue Content, Is the Main Determinant of Conduction Disturbances in Human Atrial Fibrillation. EP Eur..

[29] Cunha P.S., Laranjo S., Heijman J., Oliveira M.M. (2022). The Atrium in Atrial Fibrillation—A Clinical Review on How to Manage Atrial Fibrotic Substrates. Front. Cardiovasc. Med..

[30] Gruden G., Giunti S., Barutta F., Chaturvedi N., Witte D.R., Tricarico M., Fuller J.H., Perin P.C., Bruno G. (2012). QTc interval prolongation is independently associated with severe hypoglycemic attacks in type 1 diabetes from the EURODIAB IDDM complications study. Diabetes Care.

[31] Novodvorsky P., Bernjak A., Downs E., Smith A., Arshad M.F., Oprescu A.I., Jacques R.M., Lee J., Heller S.R., Iqbal A. (2025). Electrocardiograpic responses during spontaneous hypoglycaemia in people with type 1 diabetes and impaired awareness of hypoglycaemia. Diabet. Med..

[32] Robinson R.T., Harris N.D., Ireland R.H., Lee S., Newman C., Heller S.R. (2003). Mechanisms of abnormal cardiac repolarization during insulin-induced hypoglycemia. Diabetes.

[33] Frier B.M., Schernthaner G., Heller S.R. (2011). Hypoglycemia and cardiovascular risks. Diabetes Care.

[34] Fuller W., Tulloch L.B., Shattock M.J., Calaghan S.C., Howie J., Wypijewski K.J. (2013). Regulation of the cardiac sodium pump. Cell. Mol. Life Sci..

[35] Shattock M.J., Ottolia M., Bers D.M., Blaustein M.P., Boguslavskyi A., Bossuyt J., Bridge J.H.B., Chen-Izu Y., Clancy C.E., Edwards A. (2015). Na^+^/Ca^2+^ exchange and Na^+^/K^+^-ATPase in the heart. J. Physiol..

[36] Kistamás K., Veress R., Horváth B., Bányász T., Nánási P.P., Eisner D.A. (2020). Calcium Handling Defects and Cardiac Arrhythmia Syndromes. Front. Pharmacol..

[37] Jones J., James S., Brown F., O’Neal D., I Ekinci E. (2022). Dead in bed—A systematic review of overnight deaths in type 1 diabetes. Diabetes Res. Clin. Pract..

[38] Lin Y.K., Hung M., Sharma A., Chan O., Varner M.W., Staskus G., Fisher S.J. (2019). Impaired Awareness of Hypoglycemia Continues to be a Risk Factor for Severe Hypoglycemia Despite the Use of Continuous Glucose Monitoring System in Type 1 Diabetes. Endocr. Pract..

[39] Mendez C., Kaykayoglu C.A., Bähler T., Künzler J., Lizoain A., Rothenbühler M., Schmidt M.H., Laimer M., Witthauer L. (2025). Toward Detection of Nocturnal Hypoglycemia in People with Diabetes Using Consumer-Grade Smartwatches and a Machine Learning Approach. J. Diabetes Sci. Technol..

[40] Alhaddad A.Y., Aly H., Gad H., Al-Ali A., Sadasivuni K.K., Cabibihan J.-J., Malik R.A. (2022). Sense and Learn: Recent Advances in Wearable Sensing and Machine Learning for Blood Glucose Monitoring and Trend-Detection. Front. Bioeng. Biotechnol..

[41] Gill G.V., Woodward A., Casson I.F., Weston P.J. (2009). Cardiac arrhythmia and nocturnal hypoglycaemia in type 1 diabetes—The ‘dead in bed’ syndrome revisited. Diabetologia.

